# SIX1 represses senescence and promotes SOX2-mediated cellular plasticity during tumorigenesis

**DOI:** 10.1038/s41598-018-38176-0

**Published:** 2019-02-05

**Authors:** Cristina De Lope, Samara Martín-Alonso, Jaione Auzmendi-Iriarte, Carmen Escudero, Isabel Mulet, Javier Larrasa-Alonso, Irene López-Antona, Ander Matheu, Ignacio Palmero

**Affiliations:** 1 Instituto de Investigaciones Biomédicas “Alberto Sols” CSIC-UAM, Madrid, Spain; 2 Instituto de Investigación Sanitaria Biodonostia, San Sebastián, Spain; 3 IKERBASQUE, Basque Foundation for Science, and CIBERfes, Madrid, Spain; 4 Present Address: Centro de Biología Molecular Severo Ochoa, CSIC-UAM, Madrid, Spain; 5 Present Address: Instituto de Biomedicina de Valencia CSIC, Valencia, Spain; 6 Present Address: Centro Nacional de Investigaciones Cardiovasculares, Madrid, Spain

## Abstract

Six1 is a developmental transcriptional regulator frequently overexpressed in human tumors. Recent results show that SIX1 also acts as a repressor of cell senescence, an antiproliferative response with a key role in tumor suppression, among other physiological and pathological settings. Here, we set to study the impact of SIX1 gain of function in transformation and tumorigenesis of fibroblasts, in connection with senescence. Using transcriptomic, histological, and functional analyses in murine tumors and cells of fibroblast origin, we show that SIX1 has a strong pro-tumorigenic action in this model, linked to the repression of a senescence-related gene signature and the induction of an undifferentiated phenotype mediated, at least in part, by the regulation of the stemness factor Sox2. Moreover, functional analyses with human glioma cell lines also show that SIX1 controls SOX2 expression, senescence and self-renewal in this model. Collectively, our results support a general link of SIX1 with senescence and SOX2-mediated cell plasticity in tumors.

## Introduction

Tumor formation is a multistep process that involves the acquisition of oncogenic traits and is opposed by diverse tumor suppressor mechanisms. It is well established that cellular senescence is one of such tumor suppressor mechanisms. Senescence is an antiproliferative response that controls cell balance in a variety of physiological and pathological settings, halting proliferation and triggering clearance of damaged cells^1–3^. In the context of cancer, senescence acts as an effective tumor suppressor barrier, blocking the expansion of potentially oncogenic cells in premalignant lesions^4^. We have recently shown that SIX1, a member of the SIX family of homeobox transcriptional regulators, is a negative regulator of senescence, which controls the expression of key senescence regulators such as the cell cycle inhibitor p16INK4A^5^. Work in Drosophila and vertebrate animal models has established that SIX proteins, and their cofactors of the EYA family, play a critical role during organogenesis, most notably in muscle, kidney and diverse neurosensorial structures^6^. In humans, alterations in SIX or EYA proteins are linked to the Branchio-Oto-Renal (BOR) syndrome, a developmental disease characterized by renal and otic defects^7^. In addition to its physiological role in organogenesis, it has also been shown that SIX1, and other SIX proteins, act as oncogenes in a variety of tumor types, including lung, breast, brain and colorectal tumors. SIX1 is frequently overexpressed in these tumors and it has been associated to several traits critical for tumor formation and progression, such as proliferation, angiogenesis, invasion and cancer stem cell function^8,9^. Of note, studies on SIX1 in cancer so far have focused mostly on carcinomas, and thus the knowledge about the role of Six1 in tumors of non-epithelial origin is much more limited^10,11^. Considering the role of senescence as a tumor protective barrier and the link of SIX1 to senescence in fibroblasts, we set here to investigate the role of SIX1 in fibroblast transformation and tumorigenesis, in connection with cellular senescence. To this end, we used a cellular model of oncogenic transformation and tumorigenesis based on mouse primary fibroblasts. The analysis of tumors with SIX1 overexpression indicate that the oncogenic effect of SIX1 is associated with the repression of a senescent gene signature and the induction of a dedifferentiated tumor phenotype mediated, at least in part, by the stemness regulator Sox2. Further studies with human glioma cells have confirmed these observations and clearly support the link of the pro-tumorigenic effect of SIX1 with senescence escape and SOX2-mediated self-renewal.

## Results

### SIX1 promotes fibroblast tumorigenesis

**Figure 1:**
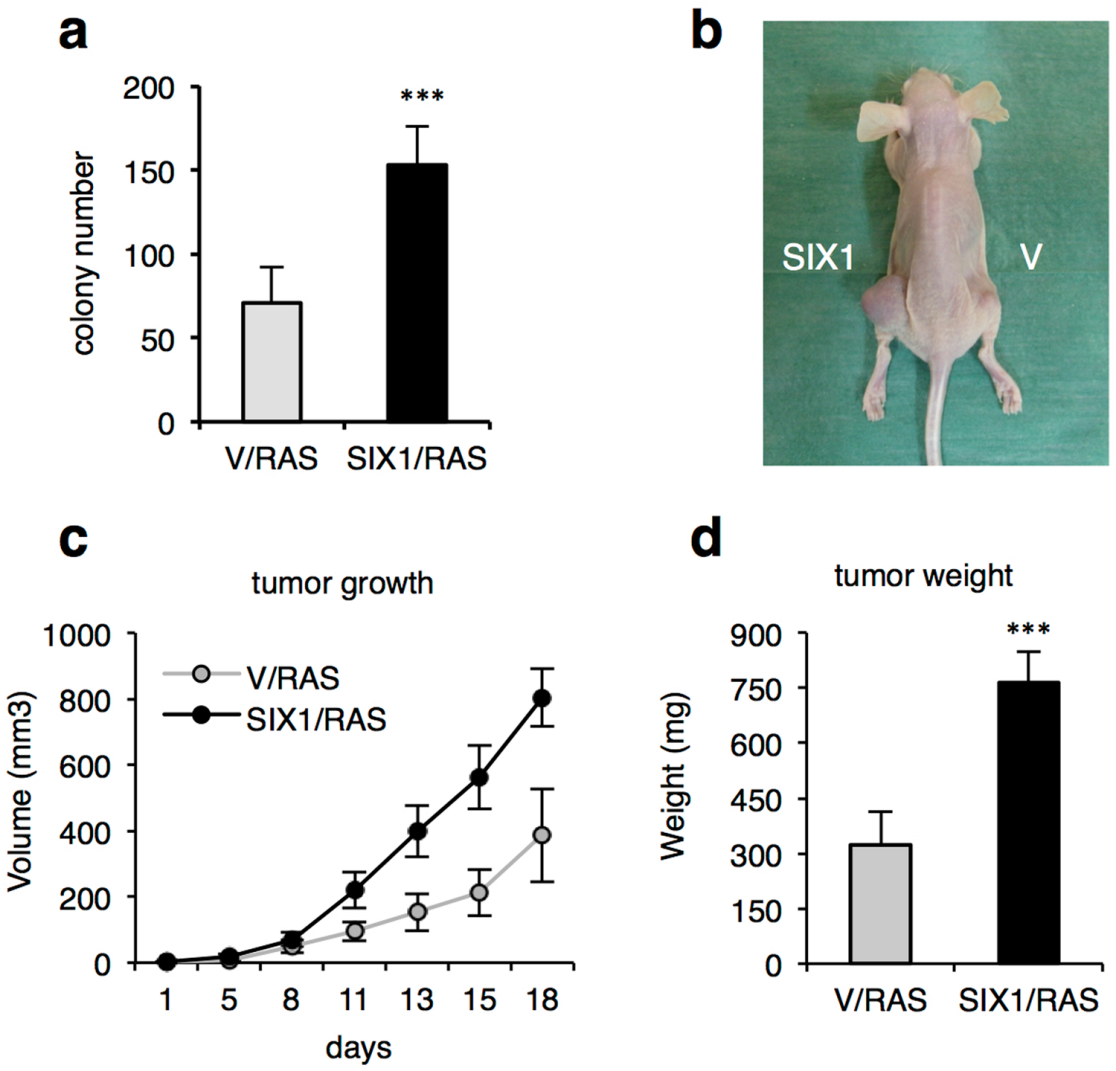
SIX1 promotes tumorigenesis in fibroblasts. (**a**) Colony formation efficiency in anchorage-independent growth assays after seeding 2 × 10^5^ cells of the indicated types (n = 3 independent assays). (**b**) Representative image of tumors formed in xenograft assays in immunodeficient mice. (**c**) Kinetics of tumor growth in xenograft assays (n = 20 tumors in 10 mice from one representative experiment of a total of two independent experiments). (**d**) Weight of tumors excised at the end of the experiment (n = 3 for each type). All the graphs show the average and standard deviation of the data.

To investigate the impact of gain of function of SIX1 in immortalization and oncogenic transformation in a genetically defined model, we have used primary Mouse Embryo Fibroblasts (MEF). These cells represent a well-established cellular model for these studies, as they can be immortalized and transformed with a small number of well-defined genetic alterations^12^. SIX1 was ectopically expressed in early passage wild-type MEF with or without expression of an shRNA against p53, using retroviral transduction. As expected, p53 knockdown was sufficient to immortalize early passage MEF. Increased SIX1 levels did not alter significantly the colony formation ability of shp53 MEF, and neither was it sufficient to allow efficient immortalization of wild-type MEF in the absence of shp53 (Data not shown). Next, immortalized fibroblasts with or without ectopic SIX1 were retrovirally infected with the activated form of the Ha-Ras oncogene, RasV12. (For simplicity, shp53/RasV12 cells are hereafter designated V/RAS, while shp53/SIX1/RasV12 cells are named SIX1/RAS, Supplementary Fig. S1). The impact of SIX1 gain of function on transformation in this model was first investigated in anchorage-independent growth assays using soft agar, which showed that SIX1/RAS cells were able to form significantly higher number of colonies than controls without SIX1 overexpression (Fig. 1a). Of note, SIX1 ectopic expression alone was not sufficient to confer anchorage independent growth in these assays (Data not shown). To evaluate the effects of SIX1 overexpression in tumorigenicity *in vivo*, V/RAS and SIX1/RAS fibroblasts, together with controls lacking RasV12 expression, were injected subcutaneously in immunodeficient mice. Both types of RasV12-expressing fibroblasts formed tumors in all cases, as expected, while immortal fibroblasts with SIX1 overexpression in the absence of RasV12 failed to form tumors, consistent with the *in vitro* data. Of note, tumors with SIX1 overexpression showed a faster growth rate and reached larger size and weight at the end of the experiment (Fig. 1b–d), indicating that SIX1 promotes tumor growth in this context. All the tumors were diagnosed as encapsulated subcutaneous fibrosarcomas by histopathological analysis. They contained cellular atypias such as hypercromatic or giant nuclei or multinucleated cells, among others, irrespective of their genotype (Supplementary Fig. S2).

### Regulation of senescence-associated genes during SIX1 tumorigenesis

**Figure 2:**
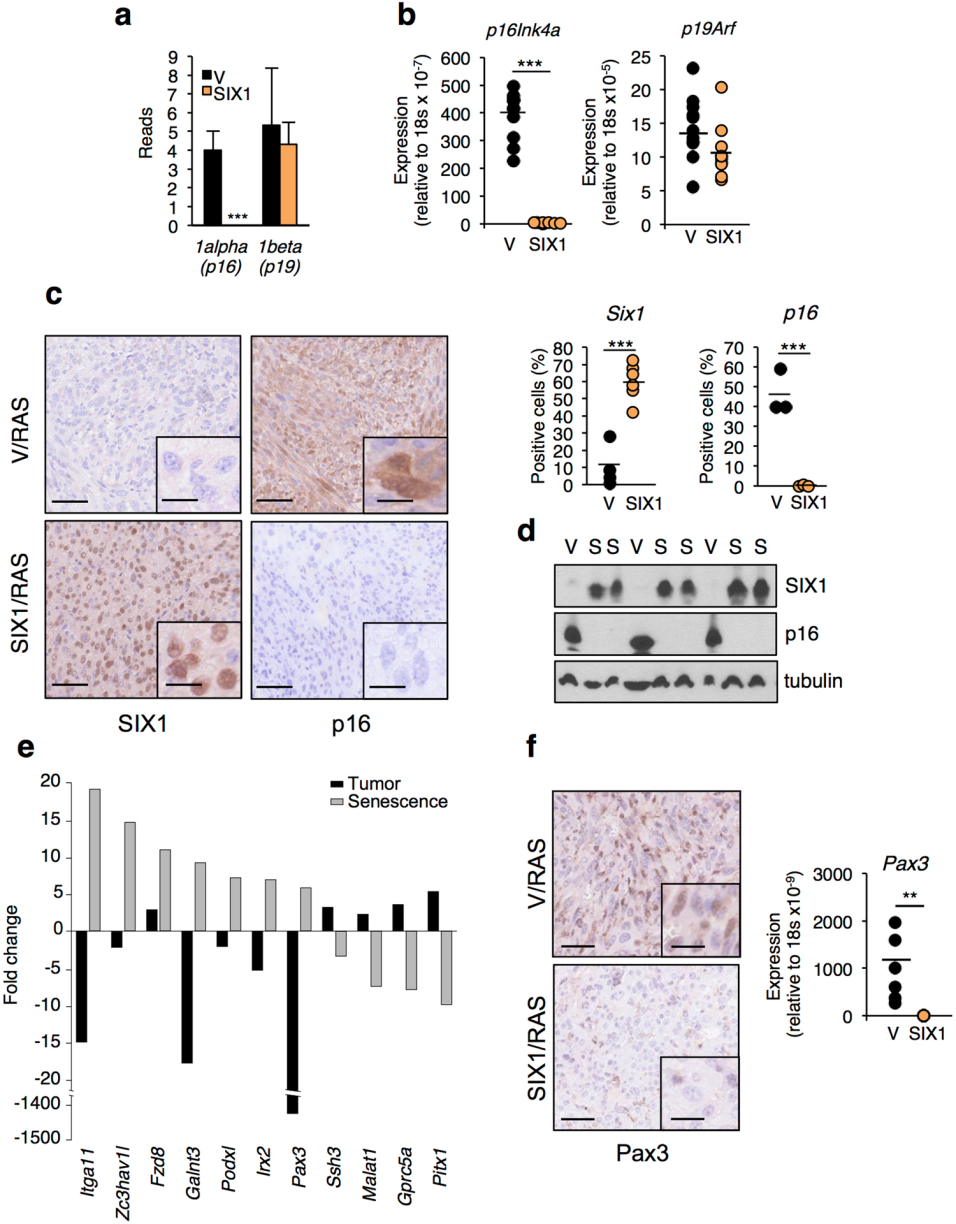
Altered expression of senescence-associated genes in SIX1-tumors. (**a**) RNASeq results for the two transcripts of the murine *Ink4a/Arf* locus obtained from exon-specific analysis in tumors with or without SIX1 overexpression. Exon 1 alpha is specific of the *p16Ink4a* transcript and exon 1 beta is specific of the *p19Arf* transcript. (**b**) QPCR analysis of the expression of *p16Ink4a* and *p19Arf* in tumors with or without SIX1 overexpression. (**c**) Immunohistochemical detection of p16Ink4a and SIX1 in the indicated tumors. The left panel shows representative stainings and the right panel shows a quantification of positive cells (V/RAS n = 3, SIX1/RAS n = 5). Scale bar, 50 µm; inset, 20 µm. (**d**) Western blot analysis of the indicated proteins in tumors with (S) or without (V) SIX1 overexpression. (**e**) Differential expression of SIX1-senescence-associated genes in the murine xenografts from this study and in senescent human fibroblasts. The graph shows the expression fold change of the indicated genes in SIX1-expressing tumors relative to control tumors based on the RNASeq results (Tumor) and sh-SIX1 senescent fibroblasts relative to control fibroblasts (Senescence, data from^5^). (**f**) QPCR analysis and representative immunohistochemistry of *Pax3* in the indicated tumors.

In order to understand the molecular basis of the increased tumorigenic phenotype of SIX1-overexpressing fibroblasts, we performed a differential expression analysis in tumors with or without SIX1 overexpression, using RNASeq. We have recently shown that SIX1 is a negative regulator of senescence^5^, and this response is a barrier against tumorigenesis^4^. Thus, our first objective was to determine if suppression of senescence could play a role in the tumor-promoting action of SIX1. To this end, we interrogated the differential expression results from the RNASeq with respect to genes associated to SIX1 in senescence^5^. Our previous results in primary human fibroblasts have shown that p16INK4A, a key effector of senescence, is one of the major downstream effectors of SIX1 in this response. SIX1 directly represses p16INK4A expression to suppress cellular senescence, and SIX1 down-regulation contributes to p16INK4A induction during senescence^5^. To assess *p16Ink4a* expression in tumors with SIX1 overexpression, we performed an exon-specific analysis of the RNASeq results to discriminate the *p16Ink4a* transcript from the *p19Arf* transcript, also encoded by the *Ink4a/Arf* locus. Of note, we found a dramatic downregulation of the *p16Ink4a* transcript, but not of the *p19Arf* transcript, in the SIX1/RAS tumors, in a reverse situation to senescence. The results from RNASeq were validated by QPCR in a larger series of tumors of each genotype (Fig. 2a,b). Consistent with the RNA results, immunohistochemistry and Western Blot analyses showed undetectable levels of p16Ink4a protein in the SIX1-overexpressing tumors, in contrast to readily detectable levels in the control tumors (Fig. 2c,d and Supplementary Fig. S2). In these assays, we also confirmed the overexpression of SIX1 in SIX1/RAS tumors by immunohistochemistry, Western Blot and QPCR. No significant changes were observed for the rest of Six proteins expressed in fibroblasts. Notably, *Eya2*, the major cofactor for SIX1, was dramatically upregulated in control tumors relative to SIX1/RAS tumors, probably reflecting a selective pressure to activate the SIX/EYA pathway in fibroblastic tumors (Supplementary Fig. S3). To obtain further insight of the relevance of SIX1-associated senescence in this context, we extended this analysis to a selection of the most significantly up or down-regulated genes in senescence triggered by SIX1 silencing (SIS)^5^. Interestingly, we observed that many of the genes most upregulated in SIS were significantly downregulated in the SIX1-overexpressing tumors from this study, and the reverse was true for SIS downregulated genes, suggesting a general inverse correlation of the SIS gene signature in SIX1-dependent senescence or tumorigenesis (Fig. 2e). Using QPCR and immunohistochemistry, we validated these results for *Pax3*, a gene upregulated in SIS and significantly downregulated in SIX1-expressing tumors (Fig. 2f). To investigate changes in senescence-related genes along the different steps of the tumorigenesis process, we also analyzed their expression in immortalized and transformed fibroblasts. In addition, we derived cell lines from tumors of both genotypes, to evaluate potential selection of features during tumor formation. QPCR and Western Blot in this set of cells revealed different patterns of expression for the selected senescence-related genes (Supplementary Fig. S4). *p16Ink4a* expression was already dramatically reduced in immortalized and Ras-transformed SIX1-expressing cells before tumor formation and this pattern was retained in tumor-derived cell lines. In contrast, expression of *Pax3* became readily detectable only after RAS expression in control cells, but not in SIX1-overexpressing cells. Collectively, these results indicate that SIX1-senescence genes display a reverse pattern of expression during tumorigenesis and support the notion that blockade of senescence contributes to the oncogenic effect of SIX1 in this model.

### Regulation of stemness and differentiation genes

**Figure 3:**
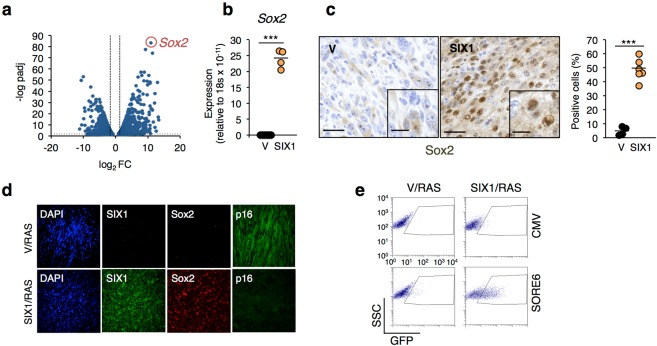
Upregulation of Sox2 in SIX1-overexpressing tumors. (**a**) Volcano plot of RNASeq results. FC, expression fold change in SIX1-tumors relative to controls; -log padj, minus logarithm of the adjusted p value. The horizontal dotted line indicates padj = 0.05, the vertical dashed lines indicate FC 2 and −2. (**b**) QPCR analysis of *Sox2* expression in tumors with (SIX1) or without (V) SIX1 overexpression, (V/RAS n = 4, SIX1/RAS n = 6). (**c**) Immunohistochemical detection of Sox2 in the indicated tumors. The left panel shows a representative staining and the right panel shows a quantification of positive cells (n = 6 for each tumor type). Main scale bar, 50 µm; inset scale bar, 20 µm. (**d**) Immunofluorescence of SIX1, Sox2 and p16Ink4a in the indicated tumors. Scale bar, 100 µm. (**e**) Flow cytometry detection of the activity of the Sox2-responsive SORE6 cassette in the indicated fibroblasts transduced with SORE6 or empty vector (CMV).

To gain further insights into the genetic basis of the tumor phenotype caused by SIX1, we analyzed the global results obtained with RNASeq. The analysis of differentially expressed genes using a volcano plot identified *Sox2* as the gene most significantly upregulated in SIX1/RAS tumors (approximately two thousand fold increase, Fig. 3a, Supplementary Table S1). Sox2 is a master regulator of stemness and cell fate during early embryogenesis and in some adult tissues. It also has important roles in cancer, where it has been linked to promoting proliferation, invasion and maintenance of cancer stem cells^13,14^. Interestingly, SIX1 is also linked to stem and progenitor cell specification both in development and cancer (see Introduction). With this background, we considered interesting to explore in more detail the link of SIX1 to SOX2 in tumorigenesis. First, we validated the differential expression of Sox2 in tumors with or without SIX1 overexpression. QPCR analysis confirmed high levels of *Sox2* in SIX1/RAS tumors compared to undetectable levels in V/RAS tumors (Fig. 3b). Further analyses by immunohistochemistry immunofluorescence and Western Blot confirmed these results (Fig. 3c–e and data not shown) and, collectively, they clearly showed a dramatic increase in *Sox2* transcript and protein in SIX/RAS tumors. We also investigated Sox2 levels in cell lines representing different steps of our tumorigenesis experiment, as shown above for senescence-related genes (Supplementary Fig. S5). We found that *Sox2* levels were modestly elevated in immortal fibroblasts with SIX1 overexpression (near the limit of detection by QPCR), but a much more dramatic increase occurred after expression of RasV12, which now could be easily detected by both QPCR and Western Blot. The differential expression of Sox2 was further retained in tumor-derived cell lines. To test if the increased levels of Sox2 associated to SIX1 overexpression had a functional impact, we introduced in V/RAS and SIX1/RAS cells the reporter construct SORE6-GFP, which contains a SOX2/OCT4 response element linked to GFP^15^. Consistent with the previous results, FACS analysis showed a significant increase in the activity of the SORE6-GFP reporter in SIX1/RAS cells, confirming that SIX1 overexpression in transformed fibroblasts is accompanied by a significant increase in Sox2 levels and activity (Fig. 3e and Supplementary Fig. S6).

**Figure 4:**
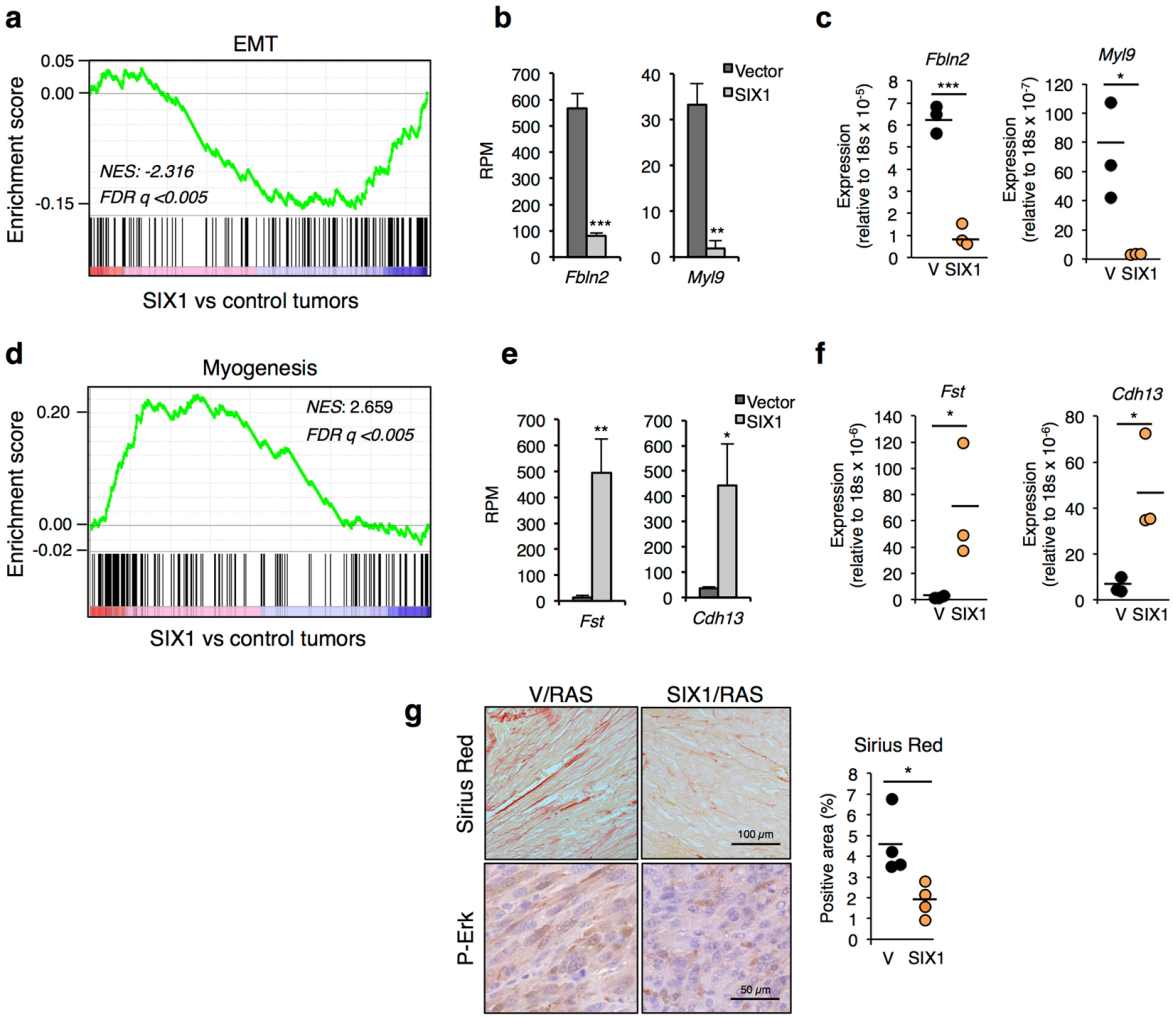
De-differentiation in SIX1-overexpressing tumors. (**a**) GSEA enrichment plot for the geneset “Epithelial Mesenchymal Transition” from the Hallmark collection. NES, normalized enrichment score; FDR, false discovery rate. (**b**,**c**) RNASeq results (Reads per million) (**b**) and QPCR analysis (**c**) of *Fbln2* and *Myl9*, two genes present in the leading edge in the analysis shown in a. (**d**) GSEA enrichment plot for the geneset “Myogenesis” from the Hallmark collection. (**e**,**f**) RNASeq results (Reads per million) (**e**) and QPCR analysis (**f**) of *Fst* and *Cdh13*, two genes present in the leading edge in the GSEA analysis shown in d. (**g**) Representative Sirius Red staining (top, quantification on right panel) and P-Erk immunohistochemistry, used here as a cytoplasmic marker (bottom) of the indicated tumors.

Given the role of Sox2 as key regulator of stemness, next we interrogated our differential expression data for further indications of changes in the differentiation state in SIX1 tumors. Interestingly, Gene Set Enrichment Analysis (GSEA) using the Hallmark collection identified significant enrichments in several categories related to differentiation, such as Myogenesis and Adipogenesis (positive correlation) or Epithelial Mesenchymal Transition (negative correlation) (Fig. 4a,d and Supplementary Table S2). A similar analysis using the Gene Ontology collection also identified several genesets related to muscle among the most significantly positively correlated terms (Supplementary Table S2). Notably, this expression profile associated to SIX1 in tumors recapitulates to some extent the physiological role of SIX1 during differentiation, since SIX1 has been implicated in both myogenesis^16^ and adipogenesis^17^. To validate this differentiation-related gene signature, we analysed by QPCR a selection of genes included in the leading edge of the categories Myogenesis (*Cdh13* and *Fst*) and Epithelial Mesenchymal Transition (*Myl9* and *Fbln2*), confirming the downregulation of mesenchymal markers and induction of muscular lineage markers in SIX1/RAS tumors (Fig. 4b,c,e,f). In support of these conclusions, RNASeq results showed that additional mesenchymal markers, such as *Thy1*, *Snail*, *Meox1* or *Fn1* were clearly down-regulated in SIX1/RAS tumors, while the cancer stem cell marker *Prom1* (also known as *CD133*) and markers of epithelial or other cell types such as *Ocln, Cldn1, Pkp1*, were clearly induced in these tumors (Supplementary Fig. S7). Interestingly, the histological analysis of SIX1/RAS tumors also indicated features consistent with de-differentiation and loss of mesenchymal phenotype. First, Sirius Red staining (Fig. 4g) indicated a reduced presence of collagen-rich stroma in SIX1 tumors, consistent with a less fibrogenic phenotype. Also, while control tumors contained predominantly cells with fibroblastic elongated morphology that formed bundles, SIX1/RAS tumors contained mostly cells with rounded, de-differentiated morphology, which were distributed more randomly (Fig. 4g). Collectively, these results indicate enhanced cellular plasticity in SIX1-expressing fibrosarcomas, as shown by the activation of Sox2 and additional markers of stemness or alternative cell linages, and the concomitant loss of mesenchymal markers.

To evaluate if the differentiation phenotype linked to SIX1 in our experimental tumors could also be observed in human mesenchymal tumors, we interrogated gene expression data from soft tissue sarcomas included in the TCGA database. Using the whole set of soft tissue sarcomas, we found a significant inverse correlation of *SIX1* with *MYL9* and *FBLN2*, two of the mesenchymal genes most significantly downregulated in the mouse SIX1-expressing tumors, and a tendency to positive correlation with *SOX2*. Interestingly, the subset of myxoid liposarcomas, characterized by highly frequent SIX1 overexpression (95% of tumors), also showed strong inverse correlation between *SIX1* and the mesenchymal markers *MYL9* and *FBLN2*, although no clear correlation with *SOX2* was observed in this case (Supplementary Fig. S8).

### Sox2 contributes to the oncogenic activity of SIX1 *in vitro*

**Figure 5:**
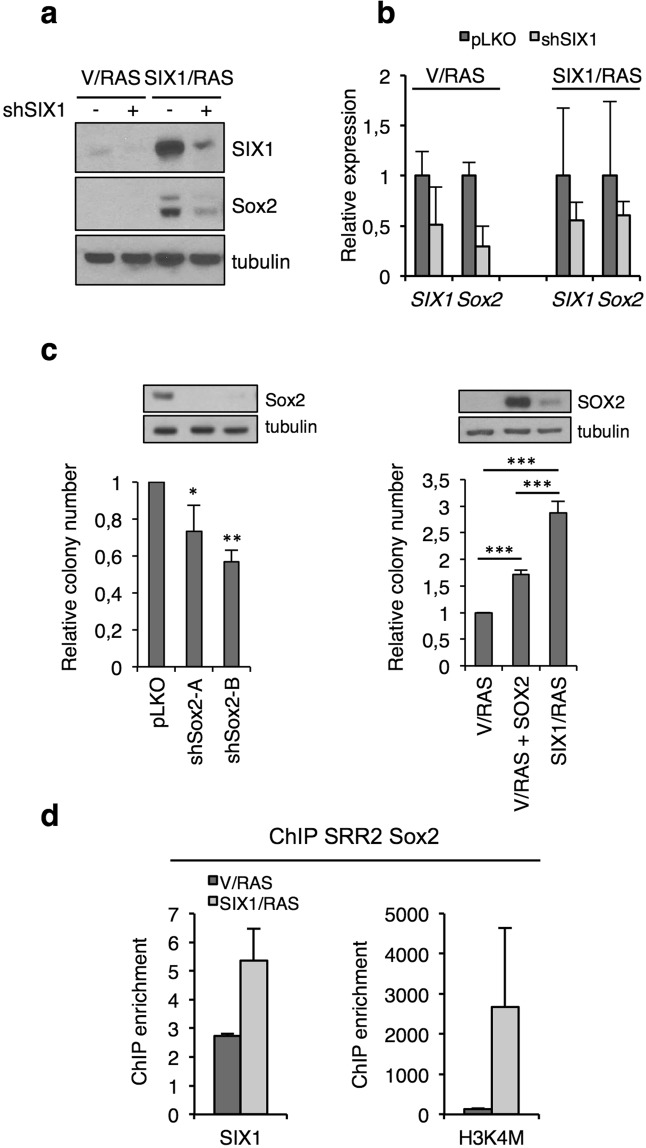
Sox2 is regulated by SIX1 and contributes to its oncogenic activity *in vitro*. (**a**) Western blot analysis of SIX1 and Sox2 in the indicated fibroblasts with or without expression of shSIX1. (**b**) QPCR analysis of *SIX1 and Sox2* expression in shSIX1 fibroblasts, relative to vector-infected cells, n = 2. (**c**) Colony formation efficiency in anchorage-independent growth assays of the indicated cell types (n = 3 except for SIX1/RAS and shSox2-A, n = 2). Western blots on top of the graphs show SOX2 levels in each cell type. (**d**) Chromatin immunoprecipitation (ChIP) of ectopic SIX1 and the histone mark H3K4me3 in the SRR2 enhancer of the Sox2 locus. The data shows enrichment of binding of the indicated antibodies relative to non-specific IgG (n = 2).

To characterize further the functional link between SIX1 and SOX2 in fibroblastic tumors, we used lentiviral shRNA to silence SIX1 in V/RAS and SIX1/RAS cells. Efficient knock-down of SIX1 resulted in down regulation of Sox2, as shown by Western Blot and QPCR, suggesting a direct link between SIX1 and Sox2 (Fig. 5a,b). Given the oncogenic role of Sox2 in different tumor types^14^, we hypothesized that Sox2 could contribute to the increased tumorigenesis of SIX1-expressing transformed fibroblasts. To test this notion, we determined the impact of the manipulation of SOX2 in anchorage-independent growth assays. Silencing of Sox2 in SIX1/RAS cells, using two independent shRNAs, caused a significant reduction in the number of soft agar colonies. Conversely, SOX2 overexpression in V/RAS cells resulted in increased colony number, which nevertheless did not equal that of SIX1/RAS cells (Fig. 5c). These results indicate that Sox2 upregulation contributes significantly to the oncogenic effect of SIX1 in our model, even though additional factors must also play a role in this phenotype.

### SIX1 binds to a regulatory element in the Sox2 locus

Next, we tried to determine the mechanism responsible for SOX2 overexpression in tumors with elevated SIX1. Based on the role of SIX1 as a transcription factor, we asked if SIX1 was present in regulatory regions of the Sox2 locus, using chromatin immunoprecipitation. This assay revealed specific binding of SIX1 to the Sox2 SRR2 downstream enhancer but not to the upstream SRR1 enhancer (Fig. 5d and Supplementary Fig. S9). SIX1 binding to SRR2 was higher in SIX1-overexpressing cells, coinciding with increased levels of the active chromatin mark H3K4me3 in this region. The SRR2 enhancer is active in pluripotent and neural stem cells^18^, as well as in specific subpopulations of tumor cells^19^. Interestingly, it contains the sequence TCACG that matches the SIX1 consensus binding motif^20^. These results showing binding of SIX1 to the SRR2 regulatory element suggest that SIX1 could participate in transcriptional regulation of Sox2.

### SIX1 controls senescence and SOX2-mediated self-renewal in glioma cells

**Figure 6:**
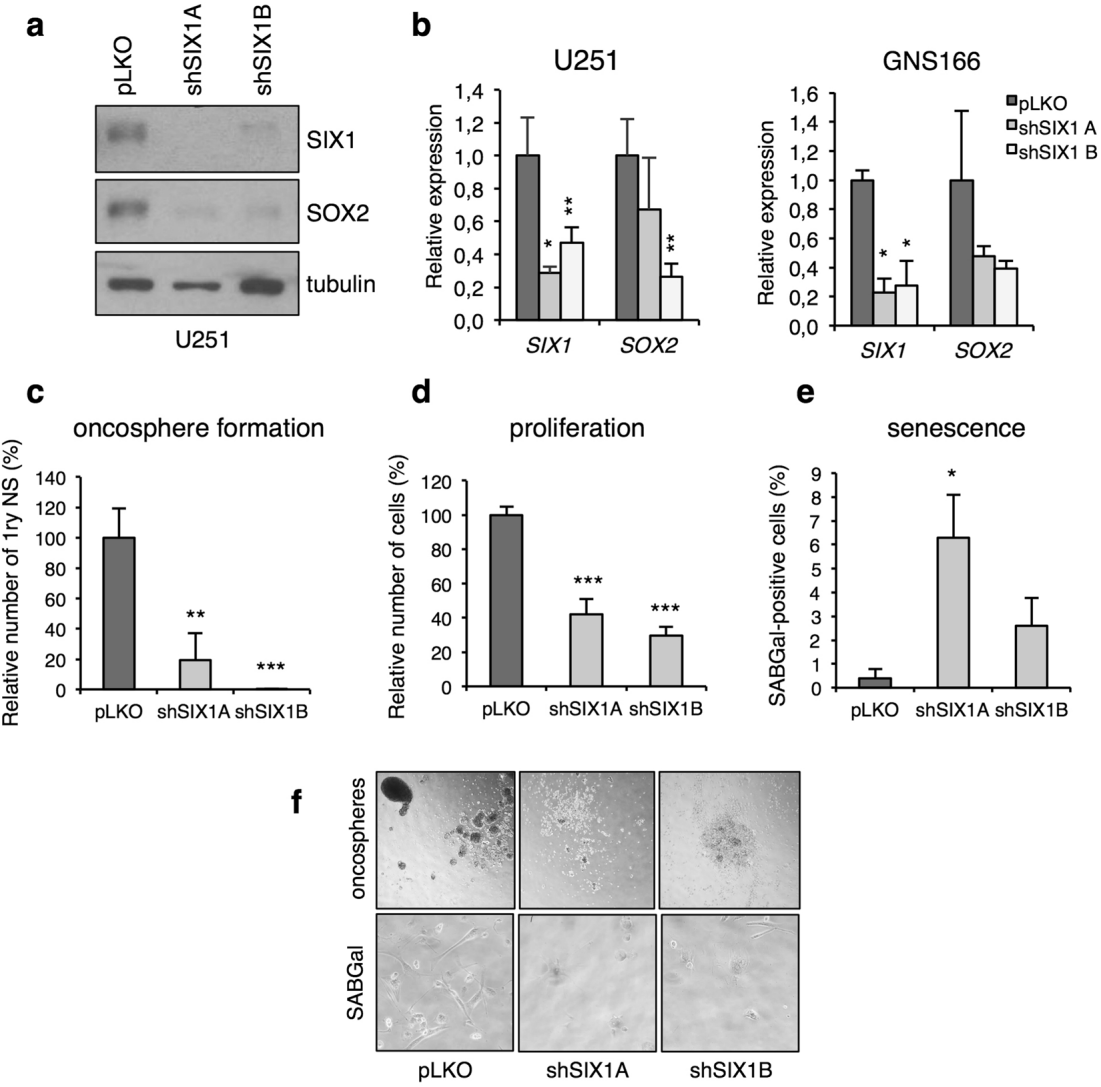
SIX1 controls SOX2 expression, proliferation and self-renewal in human glioma cells. (**a**) Western blot analysis of SOX2 in U251 cells infected with two shSIX1 vectors or control vector. (**b**) QPCR analysis of *SIX1 and SOX2* expression in U251 and GNS166 cells infected with two shSIX1 vectors, relative to vector-infected cells, n = 3. (**c**) Quantification of oncosphere forming capacity in shSIX1 U251 cells after 7 days in culture, relative to control pLKO U251 cells (n = 3). 1ry NS, primary neurospheres. (**d**) Proliferation of shSIX1 GNS166 cells. The increase in cell number (cells at day 5 relative to initial number of cells) was calculated for each cell type and the value for control pLKO cells was designated as 100% (n = 3). (**e**) Quantification of Senescence-Associated Beta Galactosidase activity (SABGal)-positive cells in GNS166 cells expressing the indicated vectors (n = 3). (**f**) Representative images of oncospheres (top) and SABGal staining (bottom) as described in panels c and e.

To establish if our results could reflect a general link between SIX1 and SOX2 in cancer, we decided to investigate this connection in a different tumor type. To this end, we focused in glioma, because SIX1 and SOX2 are frequently overexpressed in these tumors and they are both specifically enriched in glioma stem cells^21–25^. To study the link between SIX1 and SOX2 in glioma, we used the cell lines U251 and GNS166^26^, representative of differentiated and stem-like phenotypes respectively. Both cell lines express high levels of SIX1 and SOX2. Notably, silencing of SIX1, using two independent shRNAs, led to a marked reduction in SOX2 transcript and protein in both glioma cell lines, in line with the results in mouse transformed fibroblasts (Fig. 6a,b). In agreement with the key role of SOX2 in glioma cancer stem cell renewal, and the reduction in SOX2 expression caused by shSIX1, knockdown of SIX1 had a clear functional impact in glioma cells, leading to a dramatic reduction of their self-renewal capacity, as measured by their ability to form oncospheres (Fig. 6c,f). Interestingly, in keeping with its role as a senescence regulator in other cell types^5^, SIX1 silencing also caused a clear reduction in proliferation in both cell lines, which was accompanied by the induction of markers of cellular senescence, such as Senescence-Associated Beta Galactosidase activity and the acquisition of an enlarged morphology (Fig. 6d–f and data not shown). These results indicate that SIX1 controls SOX2-mediated self-renewal, proliferation and senescence in glioma cells, and they support the existence of a general link between SIX1 and SOX2 in tumors.

## Discussion

We have recently shown that the homeoprotein SIX1 is a negative regulator of senescence^5^, an intrinsic tumor-suppressive response^2,4^. Moreover, evidence from animal models and human tumors suggests that SIX1 can act as an oncogene in different types of cancer^8,9^. With this background, we have analysed here the contribution of SIX1 to fibroblastic tumors and its link to senescence, using a cellular model for immortalization and oncogenic transformation based on primary mouse fibroblasts. The tumor-suppressive role of senescence is mediated by a proliferation block in potentially oncogenic cells within premalignant lesions, which halts their progression to full-blown malignant tumors. This antiproliferative effect can be reinforced by the immune–mediated clearance of senescent cells^2^. Consistent with this tumor-suppressive effect, ablation of senescence is considered a prerequisite for tumor formation. Our transformation and tumorigenesis assays with mouse fibroblasts show a clear protumorigenic effect of SIX1, in line with the evidence in other cancer types^8,9^. Interestingly, the transcriptomic analysis of these tumors has revealed the reverse regulation of a gene signature associated to SIX1-mediated senescence, which includes p16Ink4a, a key mediator of senescence caused by SIX1 loss or other triggers^2,5^. These findings underscore the relevance of senescence as a tumor protective mechanism. Moreover, they are consistent with the notion that, in addition to other well-established functions of SIX1 in tumors such as promoting proliferation, angiogenesis and invasion, blunting of senescence may be an important component of the pro-tumorigenic action of SIX1.

In addition, our study shows that SIX1 promotes stem-like or undifferentiated phenotypes in fibroblastic and brain tumor cells, linked to the regulation of the stemness factor Sox2. This protein plays a key role in the control of pluripotency, self-renewal, proliferation and cell fate in early development and in some adult stem cells. Notably, SOX2 also plays a similar role in the context of tumors, where it has been linked to maintenance and self-renewal of cancer stem cells^13,14^. Likewise, it has been reported that SIX1 is enriched in cancer stem cells in tumors such as breast carcinoma, glioma and Wilms tumor, and it can promote the acquisition of cancer stem cell features, associated to activation of the TGF-ß or Wnt pathways^25,27,28^. The direct link to SOX2 shown here identifies a novel mechanism by which SIX1 could contribute to self-renewal in cancer stem cells. SOX2 has been associated to tumor initiating cells in a variety of human tumors including mesenchymal^29–31^ and brain^21–23^ tumors studied here, where it influences proliferation, tumorigenicity and self-renewal. In line with our observations, inspection of datasets from the TCGA collection reveals a significant correlation between SIX1 and SOX2 expression in different tumor types, including glioma, soft-tissue sarcoma, prostate and esophageal cancer (Supplementary Fig. S8). Collectively, these results suggest that SIX1 could play a widespread role in the regulation of SOX2-mediated stem phenotypes in cancer. Interestingly, previous studies have also pointed to a possible link between Six1 and Sox2 in development, during formation of neurosensory structures in the inner ear and the olfactory epithelium. Overlapping expression patterns have been reported for both proteins, but a direct link has not been unambiguously demonstrated in this context^32–34^. Taking together these observations and our current results, it is feasible that the link SIX1-SOX2 we have identified in tumors might recapitulate physiological regulatory circuits in action during organogenesis.

Our results indicate that SIX1 binds to the Sox2 SRR2 enhancer, suggesting that SIX1 might regulate Sox2 transcription in mouse cells. The significant enrichment in SIX1 tumors of pathways that have been implicated in SOX2 regulation, such as mTOR^23^ or Hedgehog^35^ prompted us to consider additional indirect effects. However, pharmacological inhibition of either pathway failed to have any significant impact on Sox2 expression in this model (Supplementary Fig. S10 and data not shown), further supporting the relevance of the direct transcriptional regulation of Sox2 by SIX1.

Recent results have shown a link between senescence and cellular plasticity or regeneration in different settings^36–40^. These reports suggest that senescent cells can promote cellular plasticity in neighboring cells through paracrine mechanisms mediated by the SASP. However, the cell-intrinsic impact of senescence on cellular plasticity is less clear^40–42^. Our results open the possibility that SIX1 might play a role in coordinating both processes in tumor cells, and future work should address this interesting question.

## Methods

### Cell culture

Cells were cultured with Dulbecco’s Modified Eagle Medium (DMEM) (GIBCO, Grand Island, NY, USA), containing 10% foetal bovine serum (FBS) and 0, 5% antibiotics (penicillin and streptomycin) at 37 °C in 5% CO_2_. For soft agar colony formation assays, 2 × 10^5^ cells were resuspended in a warmed solution of complete medium containing 0,3% agarose and plated in 60 mm dishes with a solidified bottom layer of 0, 5% agarose in complete medium, in triplicate. After two weeks, colonies larger than 100 µm were counted. To derive cell lines from tumors, portions from freshly excised tumors were washed with PBS, minced with a scalpel, digested with TrypLE Express (Gibco, Waltham, Massachusetts, USA), and seeded on 100 mm dishes with DMEM/10%FBS. The day after, non-attached cells and tissue debris were removed and the attached cells were expanded. Oncosphere formation assays with glioma cells were performed as described^23^. Senescence-associated Beta Galactosidase staining was performed as described^43^.

### Cell proliferation assay

Cells were seeded in 12-well plates (2,5 × 10^3^ cells per well), recovered by trypsinization and counted with a hemocytometer at days 1, 3 and 5 after plating.

### Retroviral and lentiviral transduction

Early passage wild-type MEFs were used for viral transductions essentially as described^5^. The vectors used were pRetroSuper mouse p53 (a gift from Rene Bernards), pWZLHygro-SIX1 (generated in this study), pWZLBlast-SOX2 (a gift from Matthew Meyerson, Addgene plasmid # 26351), pLXSN-RasV12, pLKO-shSIX1^5^ and SORE6-GFP (a gift from Lalage Wakefield^15^).

### Western blot

Western Blot analysis and preparation of total protein lysates from cells in culture were performed as described^44^. 30 µg of total lysate (for cell lines) or 100 µg (for tumors) was loaded on each lane. Protein lysates from tumors, were obtained through homogenization with a pestle in RIPA lysis buffer (10 mM NaPO_4_ pH 7.2, 300 mM NaCl, 2 mM EDTA, 0.1% SDS, 1% Deoxycholate, 1% NP40). The primary antibodies used are described in Supplementary Table S3.

### Quantitative PCR

Total RNA was isolated with TriReagent (Invitrogen, Carlsbad, California, USA). Quantitative real-time PCR was performed as described^45^, using SybrGreen. Primer sequences are described in Supplementary Table S3. Human *SOX2* was analysed using the TaqMan probe human *SOX2* (Hs01053049_s1, Roche).

### Tumorigenesis assays

2 × 10^5^ cells in 100 µl of PBS were injected subcutaneously in both rear flanks of 6-week-old female athymic nude (*Foxn1*^*nu*^*/Foxn1*^*nu*^) mice. Tumors were measured every 2 days with a caliper using the formula V = A × B^2^/2, where A is the longest diameter and B is the perpendicular diameter. Fourteen days after the injection, (or when the tumors reached the maximum allowed size), the mice were euthanized and the tumors were excised, weighed and processed for immunohistochemistry assays, protein and RNA extraction, and cell line derivation. All animal experiments were performed in accordance with relevant guidelines and regulations, approved by the ethics committees of the Instituto de Investigaciones Biomédicas and the Spanish Research Council (CSIC), and authorized by the Madrid Regional Government (Reference: PROEX 362/15).

### Immunofluorescence

Immunofluorescence with cells in culture was performed essentially as described in^45^. For tumors, antigen retrieval was performed with Envision Flex Target Retrieval Solution High pH (DAKO, Les Ulis, France), blocking and incubation with primary antibodies (Supplementary Table S3) was done in DAKO diluent solution. Secondary antibodies (Donkey anti rabbit AF-488, donkey anti-rabbit AF-555, donkey anti-goat AF-546, 1:500, ThermoFisher, Waltham, MA) were used in DAKO diluent solution with DAPI diluted 1:1 with PBS. Finally, the slides were mounted with ProLong (Invitrogen, Carlsbad, California, USA) and analyzed with a confocal spectral LSM710 (Zeiss) microscope.

### Immunohistochemistry

Immunohistochemical analysis of tumors was performed essentially as described^46^. Further details are described in Supplementary Methods.

### Flow Cytometry

V/RAS and SIX1/RAS cells were transduced with the vectors SORE6-GFP and pMX-Cherry (as an infection efficiency control). Two days post infection, cells were trypsinized, washed and resuspended in PBS (4 × 10^5^ cells/200 µl) and analysed in a Cytomics FC 500 MPL cytometer for detection of GFP and Cherry fluorescence. The MXP software was used for the cytometric analysis.

### RNA Seq

Total RNA from three tumors of each genotype was used for RNASeq analysis. A detailed description of the methods used for sequencing and data analysis is included in Supplementary Methods.

### Chromatin immunoprecipitation

Chromatin immunoprecipitation was performed essentially as described in^45^. The antibodies used were: SIX1 (HPA001893, Sigma), and H3K4me3 (Ab8580, Abcam). The primers used for PCR were: GGTGGTCGTCAAACTCTGCTAATT, AGAGTCTCGGAGAATGCCCT (SRR1) and ATTTATTCAGTTCCCAGTCCAAGC, CCCTCTCCCCCCACGC (SRR2).

### Statistical analysis

Statistical significance was calculated using unpaired two-tailed Student’s t-test (***P < 0.001, **P < 0.01, *P < 0.05).

## Supplementary information

Supplementary Information

Supplementary Table S1

Supplementary Table S2

## Data Availability

The RNASeq data generated in this study has been deposited at Gene Expression Omnibus (GEO) with the accession number GSE113385.
