# Supplementary material for: SIX1 represses senescence and promotes SOX2-mediated cellular plasticity during tumorigenesis
Source: Sci Rep. 2019 Feb 5;9:1412. doi: 10.1038/s41598-018-38176-0 (PMC6363751; doi:10.1038/s41598-018-38176-0)
Supplement: Supplementary file 1 — Supplementary Information [file 41598_2018_38176_MOESM1_ESM.pdf]

SUPPLEMENTARY INFORMATION

SIX1 represses senescence and promotes SOX2-mediated cellular plasticity during tumorigenesis.

Cristina de Lope, Samara Martín-Alonso, Jaione Auzmendi-Iriarte, Carmen Escudero, Isabel Mulet, Javier Larrasa-Alonso, Irene López-Antona, Ander Matheu, Ignacio Palmero

SUPPLEMENTARY METHODS

Immunohistochemistry

Immunohistochemical analysis of tumors was performed using an automated immunostaining platform (Ventana Discovery XT, Roche). Antigen retrieval was performed with CC1m buffer pH8.6 (Roche), and endogenous peroxidase was blocked with 3% hydrogen peroxide. The primary antibodies used are described in Supplementary Table 1. The horseradish peroxidase-conjugated secondary antibodies used were: rabbit anti-rat, (BA-4001, Vector) and mouse anti-rabbit (ab133469, Abcam), with visualization systems when needed (OmniRabbit, Ventana, Roche). Diaminobenzidine (DAB, Chromomap DAB, Roche) was used as a substrate and slides were counterstained with hematoxylin. Slides were digitally acquired with a slide scanner (AxioScan Z1, Zeiss) using the Zen Software (Zeiss). For Sirius Red staining, sections were incubated with Picro-Sirius red (1 mg/ml, Sigma) for one hour, washed twice with acidified water and dehydrated in three changes of 100% ethanol followed by two washes in xylene, and mounted in a resinous medium.

RNA Seq

Three tumors of each genotype were used for RNASeq analysis. For library construction, 1 µg of total RNA (RNA Integrity Number range 7,9 to 9,7, Agilent 2100 Bioanalyzer) was used. PolyA+ fraction was purified and randomly fragmented, converted to double stranded cDNA and processed through subsequent enzymatic treatments using Illumina's TruSeq Stranded Library Prep Kit. Adapter-ligated library was completed by PCR with Illumina PE primers. The resulting purified cDNA library was applied to an Illumina flow cell for cluster generation and sequenced on an Illumina HiSeq2500 instrument following manufacturer's protocols. Image analysis, per-cycle basecalling and quality score assignment was performed with Illumina Real Time Analysis software. Illumina BCL files were converted to bam format with the Illumina2bam tool (Wellcome Trust Sanger Institute, UK). Sequencing reads were aligned to the transcriptome with Tophat (Johns Hopkins University, USA), alignment data were then quantitated with HTSeq (EMBL, Germany) and differential gene expression analysis was performed with DESeq, or DEXSeq

for exon usage analysis. Enrichment analysis was performed with Gene Set Enrichment Analysis (GSEA, Broad Institute, USA, http://software.broadinstitute.org/gsea), using the GSEA Preranked tool (http://software.broadinstitute.org/cancer/software/genepattern/modules/docs/GSEAPreranked/1) with a list of genes ranked according to the formula (-log10padj x log2FC) where FC is the fold change in SIX1/RAS versus V/RAS tumors, and padj is the adjusted p value.

Pharmacological Treatment

1,3×10^5 per well in 24 well plates, or 5×10^5 cells per well in 6 well plates were used for treatment. Twenty-four hours after seeding, Rapamycin (Calbiochem, San Diego, USA) was added to the medium at the indicated concentrations and incubated for another 24 hours. Cells treated with the equivalent volume of vehicle in the same conditions were used as controls.

De Lope, Supp Fig. S1

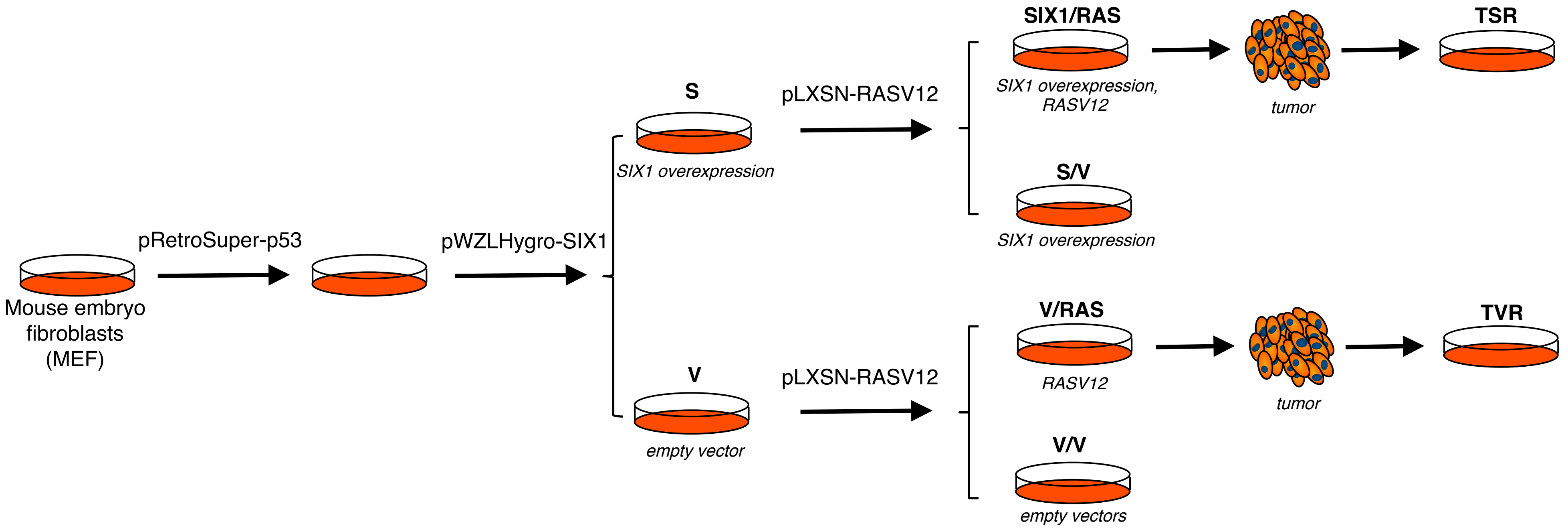

SUPPLEMENTARY FIGURE S1: Scheme depicting the generation of the different fibroblast cell lines used in this study. All the vectors indicated were retrovirally transduced. After infection with pWZLHygro-SIX1, cells were plated at low density and individual colonies were picked for further steps.

De Lope, Supp Fig. S2

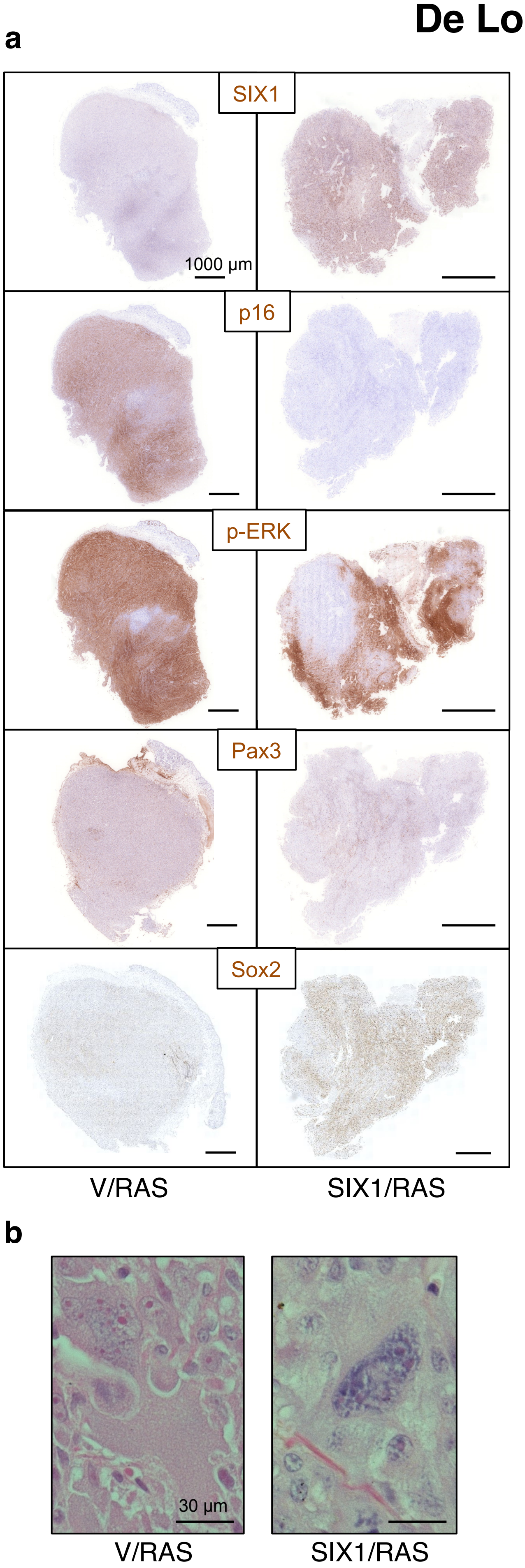

SUPPLEMENTARY FIGURE S2: Representative immunohistochemistry stainings of the indicated markers in whole V/RAS and SIX1/RAS tumors (a). Examples of cellular atypias (b).

De Lope, Supp Fig. S3

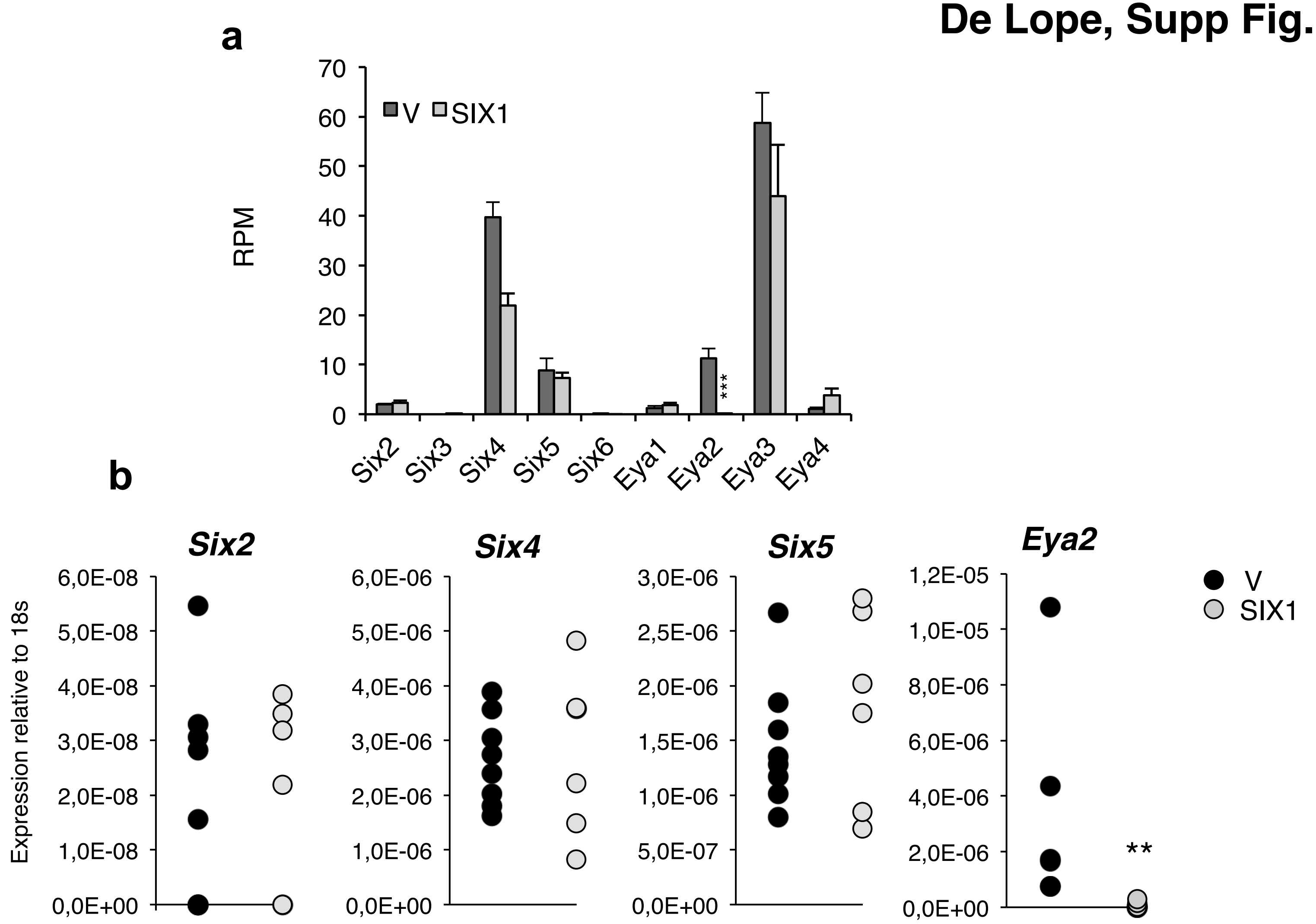

SUPPLEMENTARY FIGURE S3: Expression of members of the Six and Eya families in SIX1-tumors. (a) RNASeq results in tumors with (SIX1) or without (V) overexpression of SIX1. RPM, reads per million. (b) QPCR analysis of the indicated transcripts in the same tumors.

De Lope, Supp Fig. S4

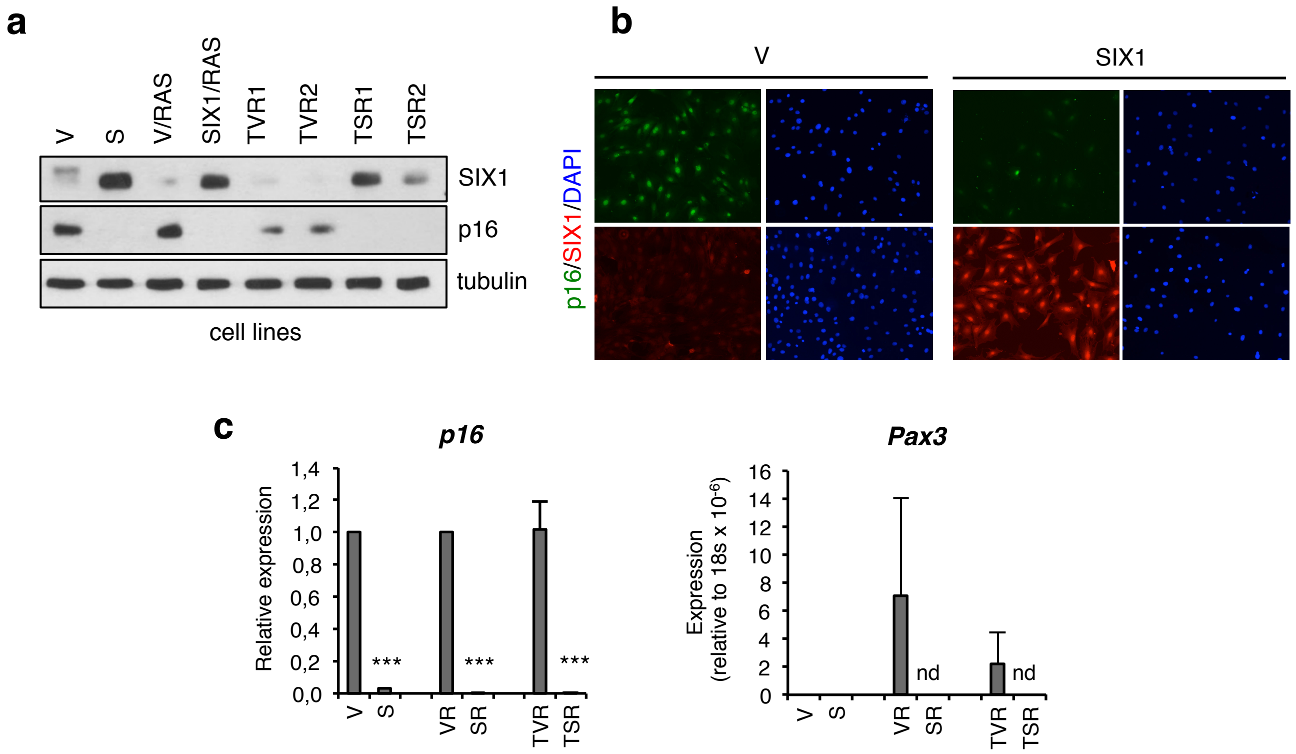

SUPPLEMENTARY FIGURE S4: Expression of senescence genes in cell lines. Western Blot analysis (a) and immunofluorescence detection (b) of SIX1 and p16Ink4a in the indicated cell lines. (c) QPCR analysis of the expression of *p16Ink4a and Pax3* transcripts in the indicated cell lines. See Suppl. Fig S1 for a key to cell line designation. TVR1, 2 and TSR1, 2 indicate cell lines derived from independent tumors of each type.

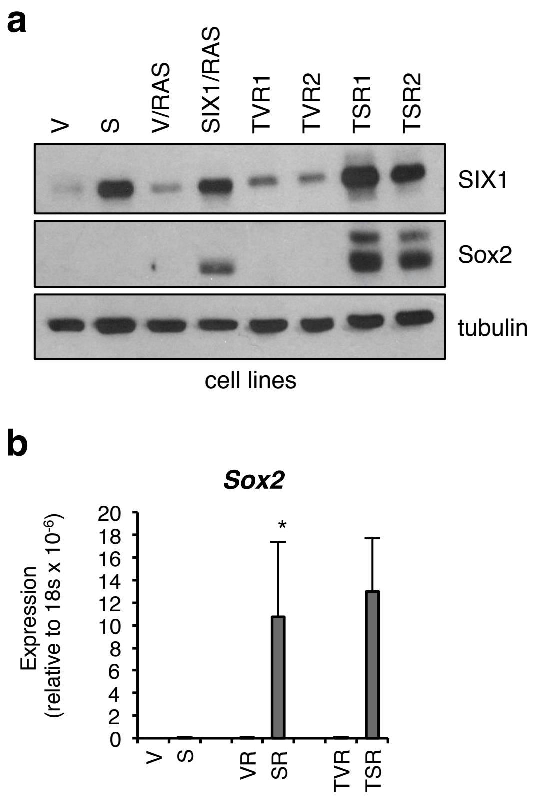

SUPPLEMENTARY FIGURE S5: Sox2 expression in cell lines. Western Blot (a) and QPCR (b) analysis of Sox2 in the indicated fibroblast cell lines. See Suppl. Figures S1 and S4 for cell line designation.

De Lope, Supp Fig. S6

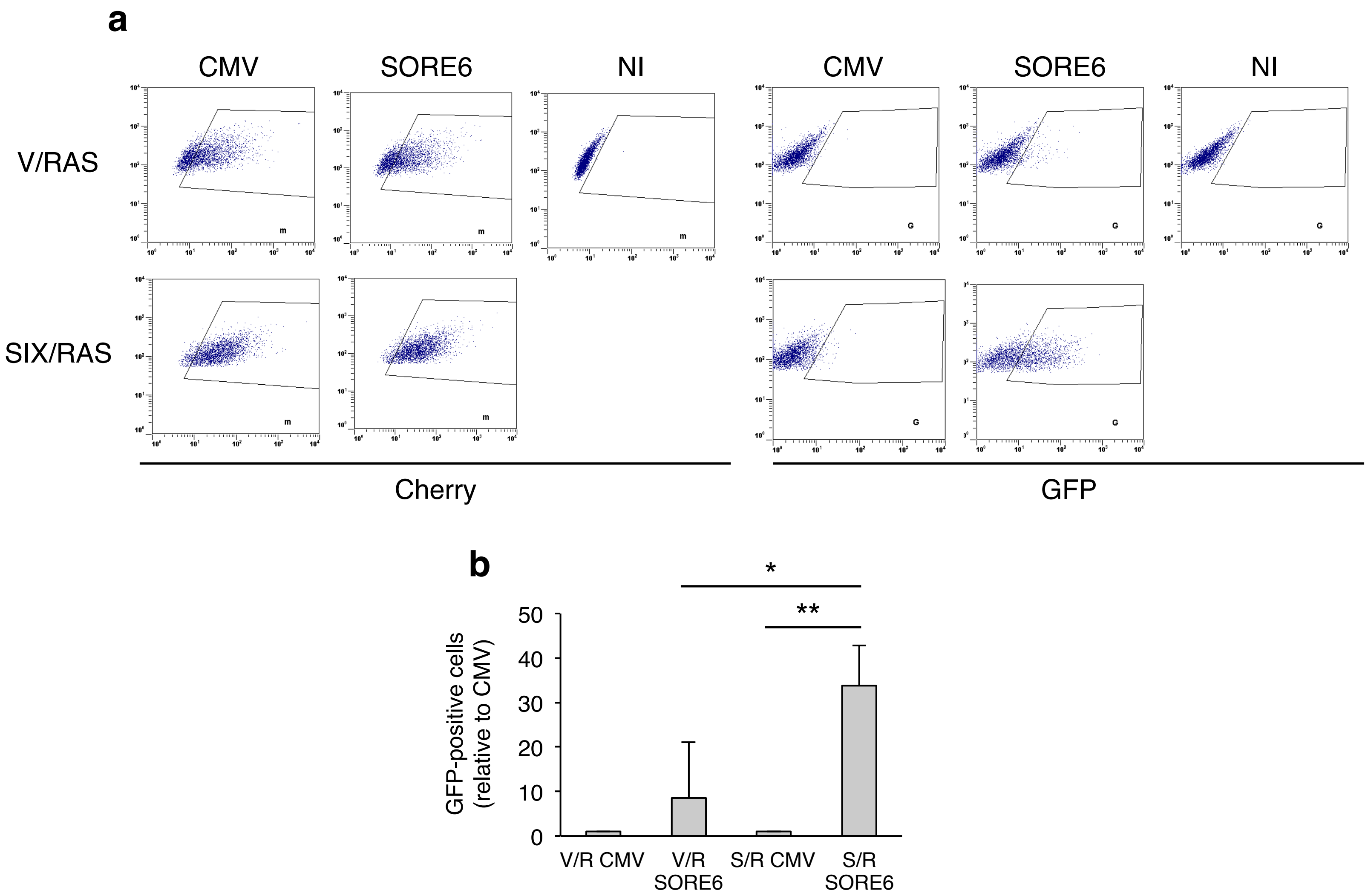

SUPPLEMENTARY FIGURE S6: Flow cytometry detection of SORE6 reporter activity (related to Figure 3e). Results from a representative experiment showing red fluorescence (driven by pMX-Cherry, infection control) and green fluorescence (driven by SORE6-GFP reporter) in V/RAS and SIX1/RAS cells infected with SORE6, its empty vector (CMV) or non-infected (NI) (a). Quantification of GFP-positive cells, relative to CMV-infected control for each cell type (n=2) (b)

De Lope, Supp Fig. S7

mesenchymal

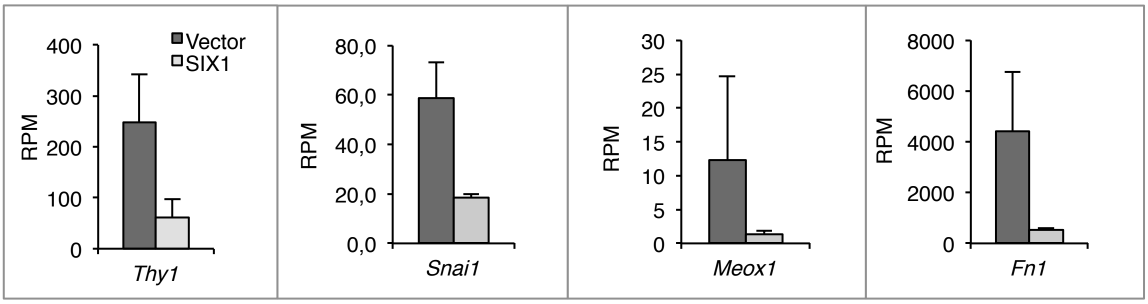

stem, epithelial

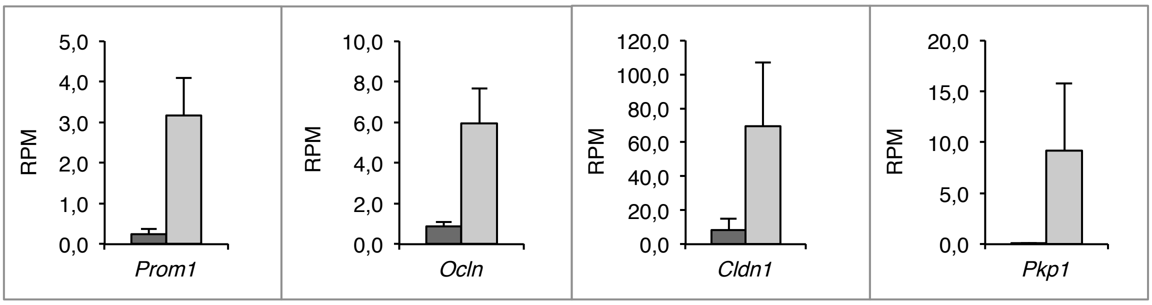

SUPPLEMENTARY FIGURE S7: RNASeq results for a set of genes related to differentiation and stemness in tumors with or without SIX1 overexpression.

De Lope, Supp Fig. S8

Esophageal carcinoma (TCGA)

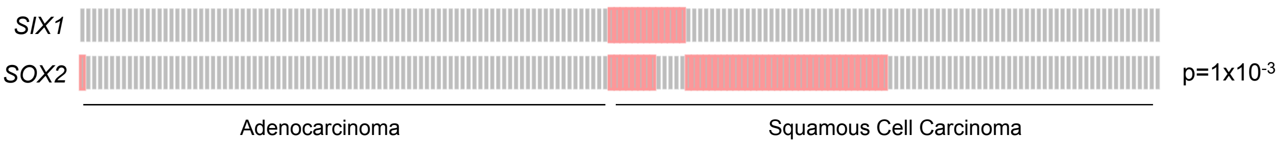

gene A	gene B	p-value	Association	Study
SIX1	SOX2	0,0000002	Tendency towards co-occurrence	Neuroendocrine Prostate Cancer (Trento/Cornell/Broad 2016)
SIX1	SOX2	0,0010638	Tendency towards co-occurrence	Esophageal Carcinoma (TCGA, Provisional)
SIX1	SOX2	0,0031826	Tendency towards co-occurrence	Head and Neck Squamous Cell Carcinoma (TCGA, Provisional)
SIX1	SOX2	0,0158450	Tendency towards co-occurrence	Brain Lower Grade Glioma (TCGA, Provisional)
SIX1	SOX2	0,0543920	Tendency towards co-occurrence	Sarcoma (MSKCC/Broad, Nat Genet 2010)
SIX1	SOX2	0,0004795	Tendency towards co-occurrence	Stomach adenocarcinoma (TCGA Nature 2014)

Soft tissue sarcoma (MSKCC)

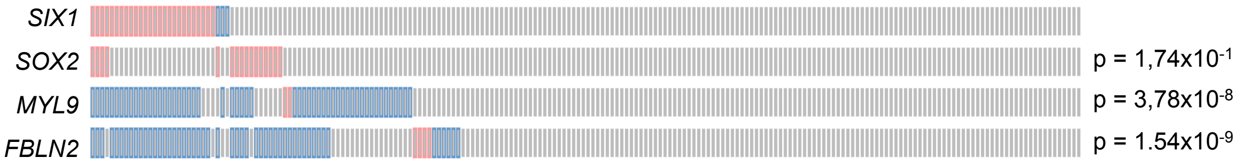

gene A	gene B	p-value	Association
SIX1	SOX2	1,74E-01	Tendency towards co-occurrence
SIX1	MYL9	3,78E-08	Tendency towards co-occurrence
SIX1	FBLN2	1,54E-09	Tendency towards co-occurrence
SOX2	MYL9	8,87E-02	Tendency towards co-occurrence
SOX2	FBLN2	1,65E-05	Tendency towards co-occurrence
MYL9	FBLN2	9,45E-08	Tendency towards co-occurrence

Myxoid liposarcoma (MSKCC)

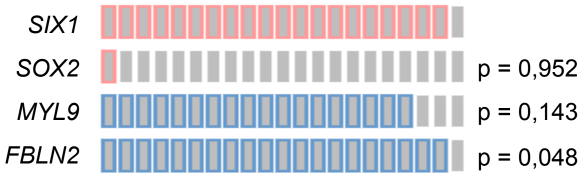

gene A	gene B	p-value	Association
SIX1	FBLN2	0.048	Tendency towards co-occurrence
SIX1	MYL9	0.143	Tendency towards co-occurrence
MYL9	FBLN2	0.143	Tendency towards co-occurrence
SOX2	MYL9	0.857	Tendency towards co-occurrence
SIX1	SOX2	0.952	Tendency towards co-occurrence
SOX2	FBLN2	0.952	Tendency towards co-occurrence

SUPPLEMENTARY FIGURE S8: Co-occurrence analysis of the expression of Sox2 and differentiation genes differentially expressed in SIX1-tumors using the BioPortal platform and data from TCGA tumor expression data base.

De Lope, Supp Fig. S9

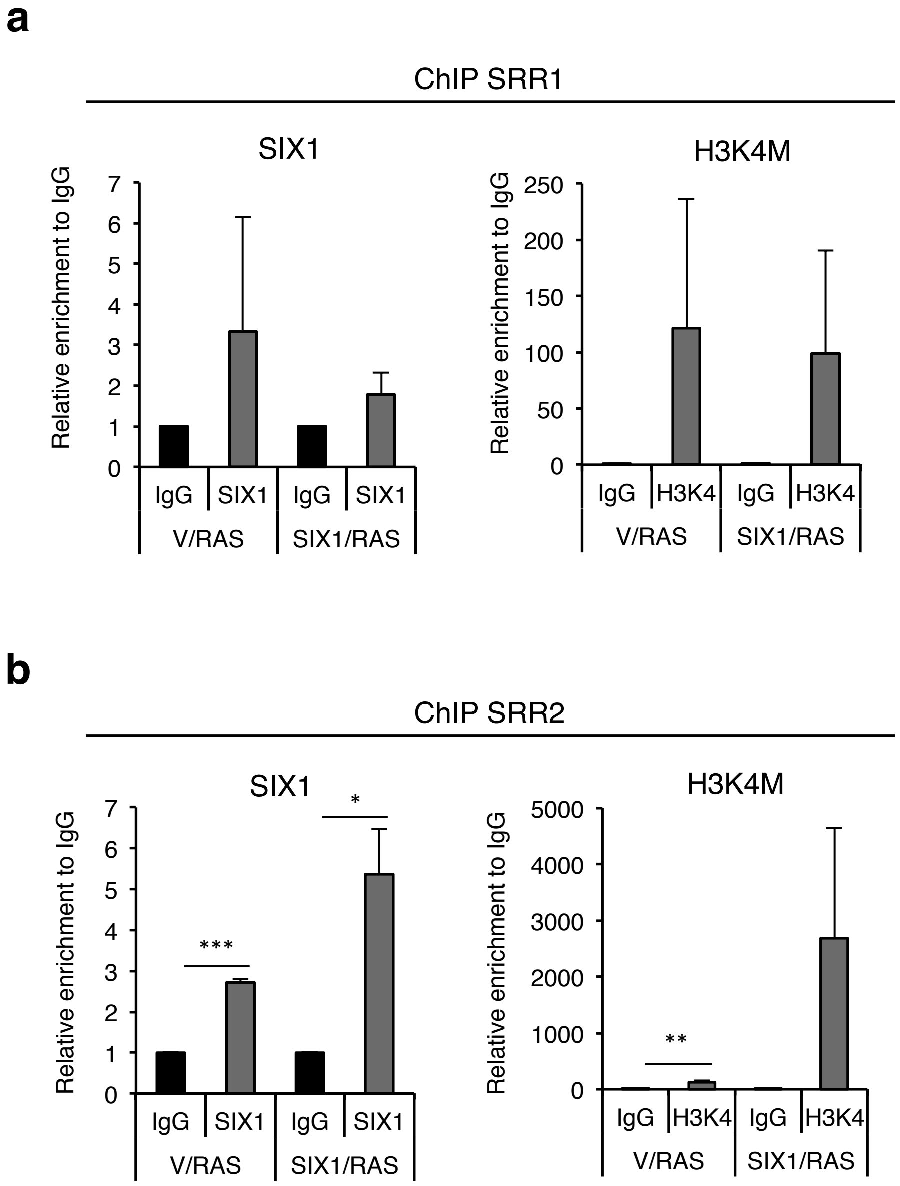

SUPPLEMENTARY FIGURE S9: Chromatin immunoprecipitation for the regulatory elements SRR1 (a) and SRR2 (b) of the Sox2 locus. Graphs show binding (antibody-bound DNA relative to input) for each antibody (SIX1 and H3K4M) using as a reference binding for non-specific antibody (IgG) in V/RAS and SIX1/RAS cells. n=2 independent experiments.

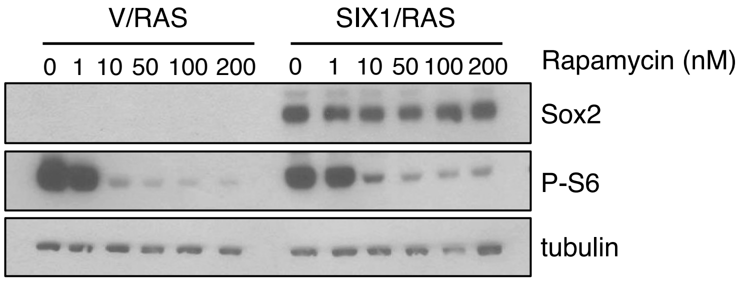

SUPPLEMENTARY FIGURE S10: Western Blot analysis of Sox2 expression in the indicated cells treated for 24h with the mTOR inhibitor Rapamycin. Phosphorylated ribosomal S6 protein (P-S6) was used as a control for mTOR activity.

De Lope, Supp Fig. S11

Figure 2

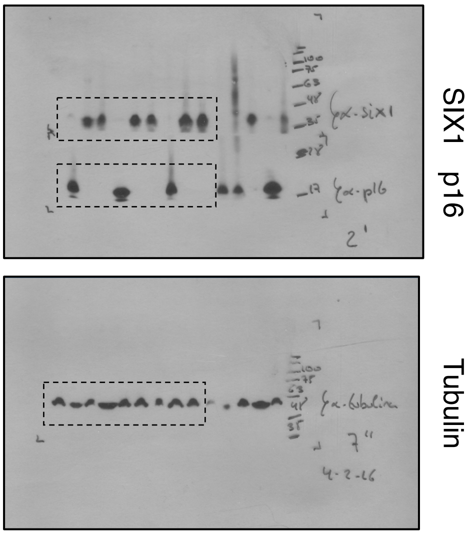

Figure 5 a

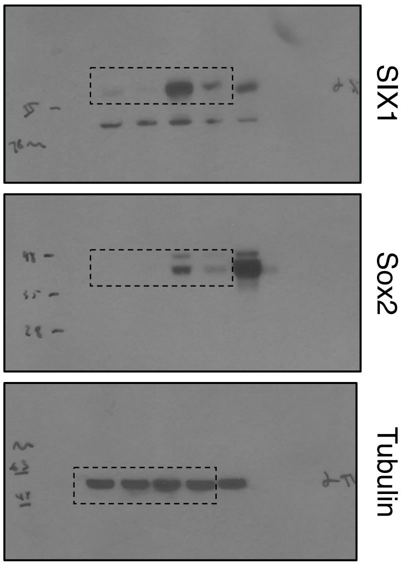

Figure 5 c (right)

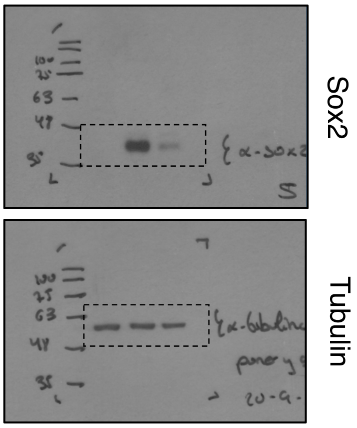

Figure 5 c (left)

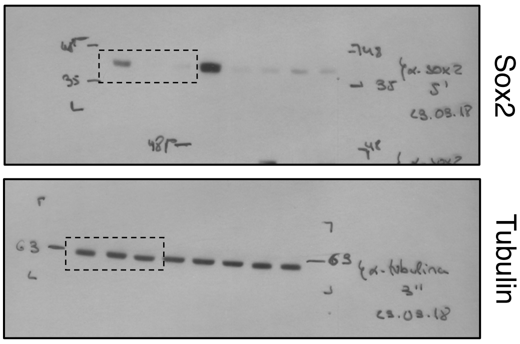

Figure 6

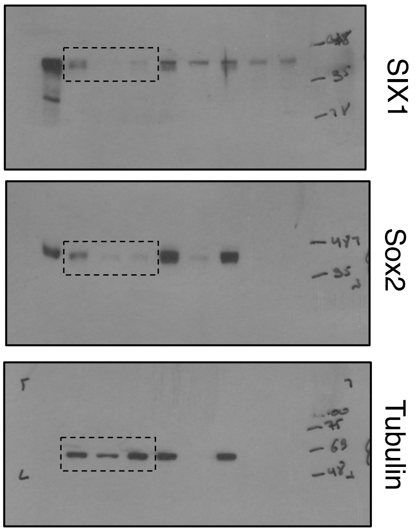

Supp Figure S4

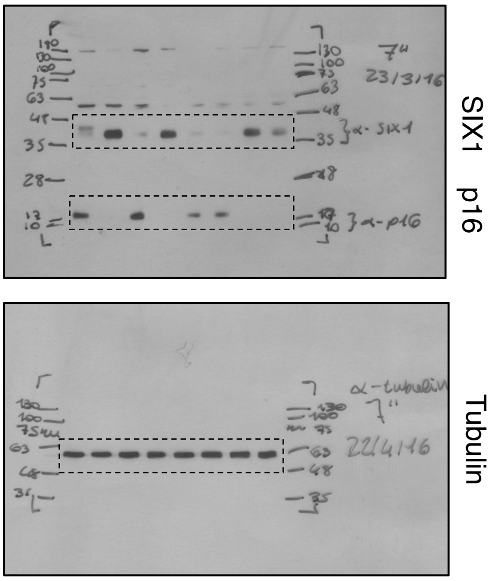

SUPPLEMENTARY FIGURE S11: Uncropped scans of immunoblots

Supp Figure S5

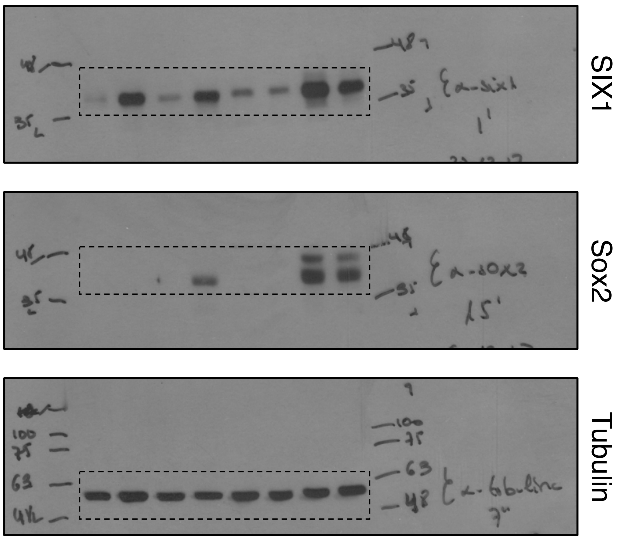

Supp Figure S10

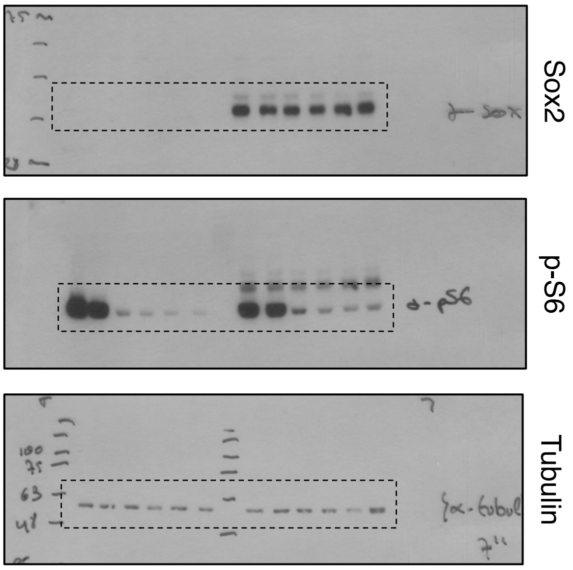

SUPPLEMENTARY FIGURE S11 (cont): Uncropped scans of immunoblots

SUPPLEMENTARY TABLE S3

PCR primers

**Transcript**	**Forward**	**Reverse**
Cdh13	CGCTTCTTCTAGTCGGGCAA	CAGGAGCACCTGGGACAG
Eya2	GGCCGGTCAAAGAGAAATAGT	AGGTCCCAGACGAACACG
Fbln2	CGGACTCTGGATTCACCGAC	TGTCTCAGGAGTCCCCGGT
Fst	CTGAGAAAGGCCACCTGCTT	TCTTCACAGGACTTTGCTTTGAT
Myl9	CTCTGCAGCAGGGAAACCC	CTTCTTGGTGGTCTTGGCCT
p16Ink4a	CGTACCCCGATTCAGGTG	ACCAGCGTGTCCAGGAAG
p19Arf	GGGTTTTCTTGGTGAAGTTCG	TTGCCCATCATCATCACCT
Pax3	GCGAGAAAAAGGCTAAACACA	CGGAGCCTTCATCTGACTG
SIX1	ACCGGAGGCAAAGAGACC	GGAGAGAGTTGGTTCTGCTTGT
Six2	CAAGTCAGCAACTGGTTCAAGA	ACTGCCATTGAGCGAGGA
Six4	GGAGCATTGGATTCTCTCCA	CCGAAGTGCTTGGGGTAAC
Six5	GCTGCAACTTCCCTCGTC	AATGGGGCTACCAGACACAG
Sox2	TAGAGCTAGACTCCGGGCGATG	TTGCCTTAAACAAGACCACGAAA

Antibodies

**Protein**	**Reference**	**Source**	**Dilution**
p16Ink4a	sc-1207	Santa Cruz	1:200 (IF),1:500 (WB)
p16Ink4a	327C	CNIO	undiluted (IHC)
Pax3	11087	DSHB	1:100 (IHC)
Phospho-Erk	9101	Cell Signaling	1:300 (IHC)
Phospho-S6	4858S	Cell Signaling	1:1000 (WB)
SIX1	HPA001893	Sigma	1:500 (IHC, WB)
SIX1	10709-1-AP	Proteintech	1:500 (IF),
SOX2	RYD-AF2018	R&D Systems	1:500 (IF, WB)
SOX2	3728	Cell Signaling	1:75 (IHC)
Tubulin	T-9026	Sigma	1:20000 (WB)

IF: immunofluorescence; IHC: immunohistochemistry; WB: Western Blot.
